# The anti-apoptotic members of the Bcl-2 family are attractive tumor-associated antigens

**DOI:** 10.18632/oncotarget.134

**Published:** 2010-08-10

**Authors:** Per thor Straten, Mads Hald Andersen

**Affiliations:** Center for Cancer Immune Therapy (CCIT), Department of Hematology, Herlev University Hospital, DK-2730 Herlev, Denmark

**Keywords:** Bcl-2, Bcl-X(L), Mcl-1, cancer vaccines, immune therapy, tumor antigens

## Abstract

Anti-apoptotic members of the Bcl-2 family (Bcl-2, Bcl-X(L) and Mcl-2) are pivotal regulators of apoptotic cell death. They are all highly overexpressed in cancers of different origin in which they enhance the survival of the cancer cells. Consequently, they represent prime candidates for anti-cancer therapy and specific antisense oligonucleotides or small molecule inhibitors have shown broad anti-cancer activities in pre-clinical models and are currently tested in clinical trials. In addition, immune-mediated tumor destruction is emerging as an interesting modality to treat cancer patients. Notably, spontaneous cellular immune responses against the Bcl-2 family proteins have been identified as frequent features in cancer patients underscoring that these proteins are natural targets for the immune system. Thus, Bcl-2 family may serve as an important and widely applicable target for anti-cancer immunotherapeutic strategies, alone or in the combination with conventional therapy. Here, we summarize the current knowledge of Bcl-2 family proteins as T-cell antigens, which has set the stage for the first explorative trial using these antigens in therapeutic vaccinations against cancer, and discuss future opportunities.

## INTRODUCTION

The use of cancer vaccines to induce anti-tumor immune responses holds huge potential to complement traditional cancer therapies. Currently around 50 different therapeutic cancer vaccines have reached phase III clinical testing (not including HPV-based clinical trials) and the FDA has recently approved the first therapeutic cancer vaccine in the US (Dendreon’s Provenge, sipuleucel-T). Moreover, more than 400 phase I trials and more than 430 phase II trials are testing therapeutic cancer vaccines in the clinic (www.clinicaltrials.gov). Cancer vaccines are designed to re-calibrate the existing host-tumour interaction, tipping the balance from tumor acceptance towards tumor control to the benefit of the cancer patient.

Immunization evolves in a stepwise fashion beginning with an innate inflammatory response at the site of danger involving mast cells, resident and/or migrant monocytes, as well as NK cells and granulocytes [1]. Their effector function, e.g. secretion of pro-inflammatory chemo-/cytokines, innate cytotoxicity and thereby release of antigen, together with their capacity of antigen uptake and migratory phenotype are prerequisites to mount an adaptive immune response. Inflammatory signals allow their migration to loco-regional lymph nodes where they either transfer the antigen to professional APC or directly encounter cognate naïve or memory T cells which upon antigen recognition get activated. Primed T cells return to circulation as memory and/or effector T cells and patrol the organism in search of the relevant targets. While T-helper cells influence the activation of B- and antigen-presenting cells as well as cytotoxic T cells (CTL) only the latter have a vital function in monitoring the cells of the body and eliminating cells that are flagged for destruction and display the appropriate antigen. This function has been firmly established for virus-infected cells. However, in addition CTL are thought to provide some degree of protection against spontaneous tumors, by virtue of their ability to detect quantitative and qualitative antigenic differences in transformed cells [2,3]. Tumorigenic alterations result in an altered protein repertoire inside the cell. Class I HLA molecules sample peptides from protein-degradation inside the cell and present these at the cell surface to CTL. Thus, it is well established that peptide epitopes derived from human tumor-associated antigens, can be recognized by CTL in the context of HLA molecules [4]. Spontaneous cancers represent approx. 80% of all cancers and obviously, the antigens potentially recognized by the immune system are basically “self” antigens. Nevertheless, it is now well documented that the immune system is in fact capable of recognizing spontaneous cancers. Thus, even in the absence of treatment, cells of the immune system in the cancer patients specifically recognize cancer cells. Moreover, CTL responsible for cancer cell recognition are capable of killing cancer cells and leaving normal cells unharmed. The molecular targets recognized – which derive from proteins present in the cancer cells encompass structures that potentially are applicable in anti-cancer vaccination strategies. A large number of such target structures have been characterized over the past more than 10 years [5]. Due to the phenomenon of “immune escape”, in which antigen-negative cancer cells avoid immune recognition, strategies have been developed that conceptually focus on specifically targeting proteins that are important for the function, survival and growth of cancer cells [6]. Likewise, the tumor microenvironment, and conventional disease management has been taken into consideration. In this regard, anti-apoptotic molecules that enhance the survival of cancer cells and facilitate their escape from cytotoxic therapies represent prime candidates for immunological intervention.

Most malignancies are characterized by defects in apoptotic signalling cascades, e.g. an overexpression of the inhibitor of apoptosis protein survivin or proteins from the Bcl-2 family (e.g. Bcl-2, Bcl-X(L), or Mcl-1). Numerous studies have demonstrated Bcl-2 over-expression in solid tumors, including melanoma, breast, colorectal, prostate, and small cell lung cancer as well as cancers of hematological origin [7-12]. Bcl-X(L) is likewise implicated in the pathogenesis of cancer and increased expression is a characteristic of acute myeloid leukemia (AML) and multiple myeloma, as well as solid cancers like bladder cancer, breast cancer, pancreatic cancer and melanoma [13]. While co-expression of Bcl-2 and Bcl-X(L) is seen in some cancers, others exhibit exclusive expression of one or the other protein. In contrast to our current understanding of Bcl-2 and Bcl-X(L), considerably less is known about the role of other anti-apoptotic Bcl-2 family members. However, elevated levels of Mcl-1 have been reported for a number of solid tumors and B-cell chronic lymphocytic leukemia (B-CLL) as well as and AML and acute lymphoid leukemia (ALL) upon relapse [13-16].

Attempts to overcome the cytoprotective effects of Bcl-2, Bcl-X(L) and Mcl-1 in cancer include several different strategies (as reviewed in [17]): (i) shutting off gene transcription, (ii) inducing mRNA degradation with antisense oligonucleotides [18], and (iii) directly attacking the proteins with small-molecule drugs [19-21]. A randomized clinical trial for metastatic melanoma comparing dacarbazine alone with dacarbazine and Bcl-2 antisense demonstrated an increase in response rates and improved survival in patients with less aggressive disease, but not in patients with more aggressive disease [22]. In addition to the above-mentioned means of targeting, immune-mediated tumor destruction is emerging as an interesting modality to treat cancer patients. Notably, during the last decade spontaneous cellular immune responses against regulators of apoptosis proteins have been identified as frequent features in cancer patients. As a result hereof, survivin-directed immunotherapy was quickly moved to the clinic [23,24] and several survivin-based vaccination trials are currently ongoing at different institutes. However, whereas survivin has been given much attention as a T-cell target, the Bcl-2 family has as yet received somewhat less attention. Here, we summarize current knowledge of Bcl-2 family proteins as T-cell antigen and discuss future opportunities.

## BCL-2

Bcl-2 is a pivotal regulator of apoptotic cell death, and it is overexpressed in many cancers [25]. Consequently, the Bcl-2 protein is an attractive target for drug design and Bcl-2 specific antisense oligonucleotides or small molecule Bcl-2 inhibitors have shown broad anti-cancer activities in pre-clinical models and are currently in clinical testing [26]. However, Bcl-2 is in addition the target of spontaneous T-cell reactivity in cancer patients [27]. Hence, analyses of the protein sequence for HLA-binding motifs and subsequent testing of blood of tumor patients revealed spontaneous T-cell reactivity against Bcl-2 in patients suffering from unrelated tumor types, i.e., melanoma, pancreatic and breast cancer, as well as AML and B-CLL. These spontaneously occurring immune responses comprised of cytotoxic effector cells. This notion could be confirmed by *ex vivo* analysis of Bcl-2 reactive T cells which were capable of killing HLA-matched tumor cells [27]. Moreover, CTL clones recognizing Bcl-2-derived epitopes efficiently killed cancer cells of different origin, e.g., colorectal cancer, breast cancer and melanoma cells [28].

Bcl-2 is implicated in cancer development, tumor progression and protection of cells from a wide range of cytotoxic insults, including cytokine deprivation, irradiation, and chemotherapeutic drugs [29]. However, although Bcl-2 is an anti-apoptotic protein a rather paradoxical role of Bcl-2 have been described, showing an association between high Bcl-2 expression and improved survival in different cancers [30,31]. Bcl-2 expression and prognosis has been correlated for breast cancer patients even if treated with chemotherapy [32]. Likewise, Bcl-2 expression has been associated with improved prognosis even among patients at very high risk for distant relapse [33]. In melanoma, it has been described that Bcl-2 expression was significantly higher in the primary tumors as compared to metastatic lesions [31]. These findings were unexpected since over-expression of Bcl-2 in tumor cell results in enhanced resistance to apoptosis *in vitro* [12]. The biological basis for the association between high Bcl-2 expression and improved survival is unclear. An explanation may be that poorly differentiated tumors depend on other prosurvival pathways, and decreased Bcl-2 expression merely is a marker of aggressive tumor behaviour rather than mechanistically associated with aggressive biology [25]. However, it could in addition suggest a pivotal role of Bc-2 specific T cells in immunosurveillance of cancer. The loss of the Bcl-2 expression during progression from primary to metastatic melanoma in patients suggests an active immune selection of the respective melanoma clones by the tumor bearing host, e.g. via a specific immune response.

## BCL-X(L)

The *bcl-x* gene is transcribed into two mRNAs through alternative splicing. The anti-apoptotic protein Bcl-X(L) is produced from the long isoform, while pro-apoptotic Bcl-X(S) is derived from the short isoform mRNA [34]. The protein product of the larger Bcl-X(L) differs from Bcl-X(S) protein by an inserted region (amino acids 126-188). The anti-apoptotic protein Bcl-X(L) plays an important role in cancer as it has been directly linked to resistance to conventional forms of therapies and poor prognosis [13]. Increased expression of Bcl-X(L) has been reported in a variety of different malignancies including AML and multiple myeloma as well as solid cancers like bladder cancer, breast cancer, pancreatic cancer and melanoma [13]. The functional inhibition of Bcl-X(L) restores the apoptotic process and renders neoplastic cells sensitive to chemical and radiation therapies, whereas manipulation of cancer cell lines to express high levels of Bcl-X(L) results in a multi-drug resistance phenotype. Thus, the attractiveness of targeting Bcl-X(L) in vaccination is based on the fact that downregulation or loss of expression of this protein as some form of immune escape would impair sustained tumor growth. The combination of immunotherapy targeting Bcl-X(L) with conventional chemotherapy appears to be particularly appealing since expression of this protein is correlated with drug resistance [35,36]. It was demonstrated that breast cancer patients, melanoma patients and pancreatic cancer patients host spontaneous HLA-restricted T-cell responses specifically against Bcl-X(L)-derived peptides [37]. In contrast, no responses were detected against Bcl-X(L) epitopes in healthy controls. Furthermore, Bcl-X(L) specific T cells not only killed target cells pulsed with the antigenic peptide but also recognized tumor cells endogenously expressing the Bcl-X(L) protein in an antigen specific and HLA-restricted manner [38]. Thus, whereas HLA-matched cancer cell lines of different origin were very effectively lysed by the Bcl-X(L) specific T cells, there was no lysis of the HLA-mismatched breast cancer cells. The killing of tumor cells of different origin underlines the universal characteristics of Bcl-X(L) as a tumor antigen. Importantly, the Bcl-X(L)-specific T cells did not only lyse *in vitro* generated tumor cell lines, but in addition lysed *ex vivo* enriched AML cells demonstrating that killing is not restricted to long-term cell lines. Since T cells and B cells normally express Bcl-X(L) following activation, Bcl-X(L) can not be considered to be a cancer-specific protein and caution is required when targeting this protein in vaccination therapies. However, the Bcl-X(L)-specific T cells did not kill purified B and T cells, which suggest that although non-malignant, B cells and T cells express Bcl-X(L), they escape recognition from Bcl-X(L) specific T cells. Similar findings have been reported for another anti-apoptotic protein, survivin. Thus, although activated B and T cells express survivin, survivin-specific CTL did not recognize and kill such cells *ex vivo* [39].

## MCL-1

The lower rates of relapse in allogeneic transplantation compared with autologous bone marrow transplantation, the striking clinical benefit of donor-lymphocyte infusions as well as the finding that human T cells can destroy chemotherapy-resistant cell lines from chronic myeloid leukemia and multiple myeloma, have prompted development of immunotherapeutic strategies against haematological cancers [40]. Among these approaches, active specific immunization or vaccination is emerging as a valuable tool to boost the adaptive immune system against malignant cells. Mcl-1 is an apoptosis-inhibiting member of the Bcl-2 family that is expressed in early monocyte differentiation. Elevated levels of Mcl-1 have been reported for a number of solid and hematopoitic cancers, e.g. CLL and in AML and ALL upon relapse [13,15,16]. In CLL patients, higher levels of Mcl-1 are strongly correlated with failure to achieve complete remission after single-agent therapy. In multiple myeloma Mcl-1 plays an important role in the survival of malignant cells [41].

Using the IFN-g ELISPOT we demonstrated that both hematopoetic and solid cancer patients host spontaneous T-cell responses against Mcl-1-derived peptides [42-44]. Thus, strong and frequent CTL responses against Mcl-1 were detected in CLL patients, melanoma patients, pancreatic cancer patients and breast cancer patients, whereas no responses could be detected in healthy individuals. Mcl-1 specific T-cell clones effectively lysed HLA-matched melanoma cells [45]. Moreover, Mcl-1-specific T cells lyse *ex vivo* enriched AML cells.

## CO-TARGETING OF THE BCL-2 FAMILY

Many anti-cancer vaccination strategies are already focusing on the combination with other immunotherapeutic strategies, e.g. the addition of cytokines or immune modulating agents. However, so far most peptide based vaccination trials have targeted only a single antigen. To maximize the impact of immunotherapy, an exciting strategy would be to co-target biologically connected proteins, e.g. the Bcl-2 family, in a multi-epitope setting. In this regard, Tanaka et al described that the co-expression of different apoptosis regulators in breast carcinoma was strongly associated with reduced apoptotic index (AI) and poor overall survival [46]. A similar association has been described in other cancers [47-49]. Thus, in most human cancers, inhibition of apoptosis is a general feature, and expression of anti-apoptosis genes, e.g. survivin and/or Bcl-2 family, may cause more pronounced antiapoptotic effects, as reflected in reduced AI. Although regulators of apoptosis proteins are up regulated in almost all cancers there may be significant quantitative divergences concerning the amount of each protein in individual patients suffering from the same disease. Previously, we have generated Bcl-2, Bcl-X(L) and survivin specific cytotoxic T cell clones and examined the killing of a panel of cancer cell lines [50]. Although all cancer cell lines were recognized and lysed by the T-cell clones the most effective lysis varied greatly among the cell lines. Thus, some target cells were killed most efficiently by Bcl-2 specific T cells, some by Survivin specific T cells and some by Bcl-X(L) specific T cells. Although this notion needs to be substantiated in large scale studies in which the clinical and prognostic significance of the CTL responses is scrutinized, it could point to a scenario in which the targeting of more than one of the targets would be beneficial in a clinical setting.

## COMBINATION WITH CONVENTIONAL THERAPY

Even few years ago the concept of combining chemotherapy – one of the side effects of which is suppression of immune function – with active immune therapy, was unheard of. However, recent data from the clinic suggests a synergistic effect of anti-cancer vaccines and chemotherapy. As an example hereof, the combination of immunotherapy with high-dose chemotherapy has been described to improve the severe immunodeficiency and leave to the induction of clinically relevant immunity in myeloma patients [51]. Notably, lympho-ablation, e.g. induced by chemotherapy, enhances the efficacy of adoptive T-cell transfer [52]. Subsequently, it may also increase a vaccination induced T-cell response. Because cytotoxic chemotherapy is widely used to treat most malignancies, integrating tumor vaccines with standard chemotherapeutic drugs is highly attractive. Several different models have explained how chemotherapy may improve subsequent or even concurrent immune therapy. Recent investigations have focused on the impact on regulatory cells [53,54]. In addition, chemotherapy might enhances tumor cell susceptibility to CTL-mediated killing [55]. Furthermore, it has been described that Antracyclins induce translocation of calreticulin to the cell surface leading to immunogenic uptake of tumor antigens [56].

Rational treatment strategies that combine tumor vaccines with cytotoxic drugs can be integrated in at least three ways as suggested by Emens and Jaffee [51]. First, chemotherapeutics can be combined with surgery and radiation to achieve a state of minimal residual disease, thereby altering the balance of the disease burden and the vaccine-induced T-cell response in favour of the T cell. Second, chemotherapy can be used to groom the local tumor microenvironment to optimally support a productive immune response. Finally, chemotherapy can be used to set the stage for a robust vaccine-induced immune response by globally altering immunoregulation within the host, subsequently permitting a robust vaccine-induced immune response.

Drug resistance is the major problem that limits the effectiveness of chemotherapies used in the treatment of cancer [57]. Drug resistance is believed to cause treatment failure in more than 90% of patients with metastatic cancer. Cancer-associated defects in apoptosis play a vital role in resistance to chemotherapy and radiotherapy [58]. An important reason for this impaired apoptosis is an over-expression of the anti-apoptotic regulators of apoptosis proteins, i.e. the Bcl-2 family [17]. Consequently, the combination of immunotherapy targeting these antigens with conventional chemotherapy appears to be particularly appealing. In a combinational therapeutic setting, conventional therapy would kill the majority of the cancer cells, leaving only cells that express high levels of antigens. Such high-expressers would be particularly vulnerable to killing by vaccination induced T cells. Thus, the synergy of these measures could potentially give a more effective treatment than the added effect of either regime alone; thereby strengthen the already described synergistic effect of anti-cancer vaccines and chemotherapy.

## CONCLUSION

It has been have reported that spontanous specific T-cell responses against the anti-apoptotic members of the Bcl-2 family are frequent in cancer patients and that these T cells are highly cytotoxic against cancer cells. Hence, Bcl-2 antigens appear to be a very attactrive target for anti-cancer immunotherapy both in hematopoetic and solid cancers. To evaluate the efficacy and safety of Bcl-2 family-based vaccinations the first phase I vaccination study have been started (from June 2010) at Herlev University Hospital, Denmark and Odense University Hospital, Denmark in which multiple myeloma patients are being vaccinated with HLA-restricted Bcl-2, Bcl-X(L) and/or Mcl-1-derived epitopes in connection with Montanide adjuvant (www.clinicaltrials.gov).

## Abbreviations used:

AI: apoptotic index
ALL: acute lymphoid leukaemia
AML: acute myeloid leukemia
B-CLL: B-cell chronic lymphocytic leukemia
CTL: cytotoxic T cells
